# Supplementary material for: Spatial epidemiological study of the distribution, clustering, and risk factors associated with early COVID-19 mortality in Mexico
Source: PLoS One. 2021 Jul 21;16(7):e0254884. doi: 10.1371/journal.pone.0254884 (PMC8294534; doi:10.1371/journal.pone.0254884)
Supplement: Attachment — Submitted filename: Answer_reviewers_08122020.docx [file pone.0254884.s001.docx]

November 25^th^, 2020

**Dr. Agricola Odoi**

**Academic Editor**

**PLOS ONE**

On behalf of my co-authors and myself I am re-submitting the manuscript entitled "Modelling the impact of health-related variables, age, migration, and socio-economic factors in the geographical distribution of early tested case-fatality risks associated with COVID-19 in Mexico”. We send the corrected manuscript and the same manuscript with changes highlighted. At the bottom of this letter we include the comments by the reviewers along with our response, with the location of the changes when appropriate.

We appreciate the opportunity of considering our work,

Sincerely yours,

Ricardo Ramírez-Aldana, Juan Carlos Gomez-Verjan, Omar Yaxmehen Bello-Chavolla, Carmen García-Peña

Correspondence

Ricardo Ramírez-Aldana

Research Division

Instituto Nacional de Geriatría

Periférico Sur No. 2767

Col. San Jerónimo Lídice,

Del. La Magdalena Contreras, D.F. 10200

ricardoramirezaldana@gmail.com

**Reviewers' comments:

Reviewer's Responses to Questions**

**Comments to the Author**

**1. Is the manuscript technically sound, and do the data support the conclusions?

The manuscript must describe a technically sound piece of scientific research with data that supports the conclusions. Experiments must have been conducted rigorously, with appropriate controls, replication, and sample sizes. The conclusions must be drawn appropriately based on the data presented.**

Reviewer #1: Partly

Reviewer #2: Partly

**2. Has the statistical analysis been performed appropriately and rigorously?**

Reviewer #1: Yes

Reviewer #2: I Don't Know

**3. Have the authors made all data underlying the findings in their manuscript fully available?**

Reviewer #1: Yes

Reviewer #2: Yes

**4. Is the manuscript presented in an intelligible fashion and written in standard English?**

Reviewer #1: Yes

Reviewer #2: Yes

**5. Review Comments to the Author**

**Reviewer #1: This paper examines the factors associated with early COVID-19 mortality in Mexico at the state level. The authors find that both internal and external migration are important, as are health risk factors (counts of diabetes, hypertension, and ICU hospitalization). Pregnancy rates are negatively associated with early COVID-19 mortality.

There is much to like about this paper. The fact that it focuses on a large, highly heterogeneous, upper middle country makes it inherently interesting, especially as Mexican health and social statistics data quality are both high. Even more importantly, the paper is meticulous in correcting for spatial autocorrelation, using generalized geographically-weighted regression techniques and, since they are estimating counts, a Poisson regression approach.**

Thank you very much for the revision. We really appreciate your time in reviewing and commenting our paper. We have modified the paper attending to all your suggestions. A point-by-point response to every raised issue is provided here.

**At the same time, I have several problems with the analysis. In rough order of descending importance:**

**1. (page 5) A key problem with analyzing the early spread of COVID-19 is that prevalence rates were poorly estimated/numbers infected were not well known. This may have extended to deaths as well, and this problem needs careful discussion.**

The reviewer is correct about this matter. We have added more about this in the discussion (**L-462**), including that for this reason, we performed the analysis thinking of the data as if it were a sample of individuals and that the fatality risks are analyzed on tested individuals (tested case-fatality risks or t-CFRs) (**L-450**), being both the analyzed number of death and sick people lower than the real numbers. Hence, care should be taken when trying to overgeneralize the obtained results. We made comparisons using the projected population in 2020 as denominator of the risks (mortality) obtaining similar results to those that we present (**L-432** and **Answer 1 Reviewer 2**).

**2. (page 5) The authors use ICU hospitalizations as a measure of case load severity. However, ICU numbers also reflect access, so that two very different effects are incorporated into a single term, making it difficult to place any meaning on the estimated coefficient.**

We have specified that ICU, intubated, and hospitalization are confounders, and that they are a measure of severity of the disease and access to services attending COVID-19 cases we wanted to control for (**L-150**). Thus, the ICU and the other confounders should not be interpreted, and it is not relevant if both effects, severity and access, are combined.

**3. ICU admissions are also endogenous: in effect, one would be better off estimating them as well in a two-stage process.**

This is true. As far as the authors know, there is not a two-stage process available for GWR. Thus, to be able to compare the results obtained with the Poisson and GWR models, we decided to include in the final model (whose selection process is later explained), residuals associated with a first Poisson model including a subset of variables corresponding to a second model, the latter being the model corresponding to the fatality risks. In the first model, we used those variables with a possible effect over ICU, namely those related with health and age. These residuals and corresponding estimated parameters do not have a meaning; however, we eliminated possible effects of variables affecting ICU also included in the model for the risks. We found this process is used in Econometrics, though care should be taken when explanatory variables are correlated; however, by construction of our selection process for the risks model, this was not a problem. The same process was performed for the other confounders **(L-219)**. Additionally, for comparison purposes, we used Principal Component Analysis (PCA) associated with the confounders and the confounders as they are, obtaining similar results. With PCA we obtained the same significant variables as those in the model with the residuals, except por diabetes (results shown in **Answer 5 Reviewer 1**), and associations with the risks in the same direction for all significant variables. When confounders were introduced as they are, the model included hypertension, and we obtained the same significant variables, except for external migration and diabetes, and the direction of association with the response is similar as the one in our model in all significant variables (**L-428**).

Model using confounders as they are (nor residuals neither PCA)

```
Coefficients:
                     Estimate Std. Error z value Pr(>|z|)
(Intercept)        -7.345222   1.451995  -5.059 4.22e-07 ***
Diabetes            0.024735   0.036172   0.684  0.49410
Obesity              0.138419   0.024436   5.665 1.47e-08 ***
ICU                  0.075423   0.036372   2.074  0.03811 *                   GDP                  0.186500   0.040214   4.638 3.52e-06 ***
Internal migration   0.123224   0.042145   2.924  0.00346 **
External migration   0.152328   0.196957   0.773  0.43928
Age 10-39           -0.054386   0.022219  -2.448  0.01437 *
Intubated            0.506521   0.107881   4.695 2.66e-06 ***
hypertension         0.047859   0.029374   1.629  0.10325
Physicians’ ratio    0.574503   0.074231   7.739 9.99e-15 ***
Cardio vascular     -0.077467   0.085188  -0.909  0.36316
Hospital            -0.009774   0.012783  -0.765  0.44449
```

Model fitted in the manuscript (using tested individuals, tested-fatality risks)

```
Coefficients:
  Coefficients:
                    Estimate Std. Error z value Pr(>|z|)
(Intercept)         -5.37567    1.27300  -4.223 2.41e-05 ***
Diabetes             0.14137    0.03335   4.239 2.25e-05 ***
Obesity              0.11534    0.01930   5.975 2.29e-09 ***
ICU (residuals)      0.20086    0.02536   7.921 2.36e-15 ***
GDP                  0.26523    0.04308   6.157 7.41e-10 ***
Internal migration   0.23680    0.03515   6.736 1.62e-11 ***
External migration   0.29916    0.12645   2.366  0.01799 *
Age group 10-39     -0.09675    0.01983  -4.879 1.07e-06 ***
Intubated (residuals)0.19779    0.03305   5.984 2.18e-09 ***
Physicians           0.62382    0.07051   8.847  < 2e-16 ***
Cardiovascular      -0.05013    0.07065  -0.710  0.47797
Hospitalized (res.) -0.04263    0.01640  -2.599  0.00936 **
```

**4. More generally, the authors include a wide range of variables as potential explanatory variables without building a model and explaining how these variables will affect mortality – whether they will do so directly or indirectly; whether the terms represent a factor that matters directly or whether they serve as proxies for omitted/unmeasurable terms. Given this absence, there can be no causal interpretation of the results.**

We have specified which variables are confounders: ICU, intubated, and hospitalization. The explanatory variables are divided into health-related variables, age, socio-demographic, economic variables, and climatic variables, all at a state level. We wanted to measure the direct effect of each health-related variable and age over the fatality risks. In terms of socio-demographic variables, we wanted to measure the impact of clustering of individuals (population density and overcrowding households), rurality, physicians’ ratio, and literacy (which was very associated with the number of cases in an analysis we performed for another country). In terms of economic variables, we wanted to measure the effect of economic importance of each state and poverty over the risks; however, as we found in our analysis, poverty, rurality, variables concerning clustering of individuals and physicians’ ratio were highly associated **(L-396)**. We also wanted to measure the direct effect of mobility, both internal and external, over the risks, being the variables we used a proxy, and now we have added the number of flights as suggested (**Answer 11 Reviewer 1)**. Finally, we wanted to measure the direct effect of climatic variables. We have rewritten the presentation of the variables in Material and Methods, including what is discussed here **(L-123).** We consider that the direct effect of each health-related variables and age is adequately measured; however, to measure the direct effect of a social or economic related variable we know it can be more difficult, since there can always be indirect effects, endogeneity, and variables heavily associated. This is also one of the reasons why we performed association analyses between all inputs in the models.

**5. (page 12) Independent variables highly correlated with one another “and less correlated with the response” were removed. A better approach might have been to have estimated principal components and used those.**

The process described before: “Independent variables highly correlated with one another and less correlated with the response were removed” was changed as follows according to suggestions of both reviewers.

Considering the suggestion to identify significant variables by fitting a Poisson model with offset for each variable (univariable models) **(Answer x Reviewer 2)** and carefully considering all statistical assumptions, particularly linear association with the transformed response **(Answer v Reviewer 2)**, the selection process was: 1) Identify and order from higher to lower (absolute value) univariable significant effects, 2) Identify which variables have good, acceptable and bad linear association with the log-transformed mortality risk, and if possible, transform variables. For instance GDP was transformed into a GDP eliminating four states: México, Ciudad de México, Jalisco, and Nuevo León, which have the greatest cities in the country (this term can be thought as the interaction between presence of the largest cities in the country and GDP). In this and the previous step, we identified that smoking was associated with lower risks, which is not logical, thus we eliminated this variable, 3) We included in the model those variables with acceptable or good linear association, and then, to avoid multicollinearity, we eliminated any variable with a VIF>10, 4) We started adding one by one significant variables, from the highest to lowest effect on the risks, and in each step we identified whether the VIF>10 to determine whether to add or not that variable to the previous model. We ended the process with a model having the variables with the greatest univariable effects, better linear behavior, and without multicollinearity (VIF<10), 5) We included residuals associated with models (as explained in **Answer 3 Reviewer 1**) concerning ICU, intubated, and hospitalization instead of the variables, 6) We checked for goodness-of-fit and model assumptions, 7) We proved the inclusion of interaction terms in the final model. The process is explained in **L-202**.

The type of analysis we intended was explanatory and not predictive, thus we wanted to be able to interpret effects over the risks. By obtaining principal components including all variables, we believe would not be able to satisfy such objective. However; following this suggestion, and as explained in **Answer 3 Reviewer 1**, we obtained principal components associated with the confounders, since for these variables we do not need any interpretation. We used the first component explaining 81% of the variability. However; since we obtained an additional significant variable with an illogical association (cardiovascular disease associated with less risks), and as we think that what was implied above is occurring here, that the effect associated with confounders is directly affected by other variables also used in the risks model, we thought that the residuals approach was more adequate. This is the approach we presented in the manuscript, but we discuss all these matters in the manuscript (**L-428**).

Analysis using the first principal component for intubated, hospital, and UCI

```
Coefficients:
                     Estimate Std. Error z value Pr(>|z|)
(Intercept)         -6.355240   1.171483  -5.425 5.80e-08 ***
Diabetes             0.052359   0.033131   1.580   0.1140
Obesity              0.169002   0.022155   7.628 2.38e-14 ***
GDP                  0.134834   0.034583   3.899 9.66e-05 ***
Internal migration   0.083284   0.038958   2.138   0.0325 *
External migration   0.544565   0.128781   4.229 2.35e-05 ***
Age 10 to 39        -0.045296   0.018689  -2.424   0.0154 *
hypertension        -0.007829   0.023385  -0.335   0.7378
Physicians           0.621224   0.067523   9.200  < 2e-16 ***
Cardiovascular      -0.173878   0.079476  -2.188   0.0287 *
PCA                  0.321060   0.033932   9.462  < 2e-16 ***
```

**6. The analysis here is a snapshot of early mortality while correcting for spatial autocorrelation. It might have been more interesting and informative to have explored early transitions using multiple time periods and capturing the interregional spread.**

We are currently working on a spatio-temporal analysis by obtaining weekly information by state concerning early and more updated information. From these analyses, we have identified that the information concerning early transitions can be problematic in certain spatio-temporal models, as spatial linear models, and we decided to be more appropriate to divide the analyses into two manuscripts.

**7. (page 6) The authors include measures of poverty and housing density. In fact, these terms are likely to matter far more for urban areas, and hence it would be better to use strictly urban measures of these terms.**

Following this suggestion, we included in our selected model interaction terms corresponding to urban proportion by state (urbanity) with poverty and urbanity with density, but we did not find any of these terms to be significant and were not included in the manuscript.

**8. (page 8, lines 157) It isn’t clear to me whether some of these measures are counts or rates. They should be defined precisely.**

We decided to better present all values as they are used in the selection process and linear models to avoid confusion, so that all analyses could be easily reproduced **(Table 1)**. Health related variables are all rates; however, for the fatality risks, we included the number of deaths and a column associated with the offset as it is used in all models.

**9. (page 5) the authors group the population into only two age classes (65+ or under 65). This is a strong assumption and adding additional age delineations in robustness check regressions would make sense.**

Thank you for your comment. Originally, we had included the group 65 years old and over to define an aging group of people. We have reanalyzed the age variable to identify whether effects of other age groups exist and how.

First, we obtained age groups in the disaggregated (individual-level) data: 0-1, 2-9, 10-19, …,80-89, and 90 years old and over and obtained the corresponding proportions by state. For each age group, we fitted univariable Poisson models with offset and obtained associations measures with the risks. We identified significant age groups and the direction of the association obtained in the models to obtain a new set of age groups: 10-39, 40-69, 70 years and over (the age group of 70-79 years was not significant in the univariable models, but according to the association measure, and to be able obtain groups including people 10 years old and over, we included it in the last group). These three groups were significant in univariable models, but only the first two had a clear linear association with the log-transformed mortality risk. Hence, these two groups were included in the first step of the selection process explained in **Answer 5 Reviewer 1** (though, the other group was later tried to be included). In our final model, only the 10-39 years old group remained, being significantly associated with lower mortality risks. All this process is explained in **L-224.**

**10. (page 16) In particular, there is no causal meaning in the finding that the percentage of pregnant women is associated with a 22-23% decrease in COVID-19 counts. The idea that pregnancy confers a “protective effect,” while possible at the individual level, seems unlikely to extend to the whole population, and the fraction of the Mexican population that is pregnant is likely too small to have a measurable effect at the aggregate level.**

Pregnancy had a clear non-linear association with the response, and in the selection process we followed now, it did not appear in the model anymore.

**11. (page 12) Although spatial autocorrelation is addressed, linkages due to air travel (both internal and international) may be important and should be considered.**

We constructed a variable consisting on the number of flights in 2019 by state using information available from the Ministry of Communications and Transport (**L-140**). This variable was significant in univariable models, though it was the variable with the lowest positive effect over the fatality risks when compared with all the other univariable models. In the selection process, this variable was eliminated, and even when added in the final model to see how it behaved, it was still not significant.

**In short, there is a fair amount to be addressed. It is important to do so: there are vast numbers of papers on COVID-19 mortality and transmission coming out, and only a few will be read or cited in a couple years. It is worth taking the care and effort to ensure that the paper falls into this elite group.**

Thank you very much. With all the suggestions provided by both reviewers, we think that our models and associated results are stronger and more reliable.

**There are minor typos that need to be corrected (for example, on line 3 of the abstract, it should read > 65, not <65; on page 4 line 75, note that the population pyramid is flattening rather than widening as Mexico ages). Beyond that, the paper is well-written, except that the table and figure titles are far too long.**

We have corrected the typo and the use of the term “widening”. We shortened the tables and figures titles.

**Reviewer #2: Manuscript #: PONE-D-20-26970

Title: Modelling the impact of health-related variables, migration, and socio-economic factors in the geographical distribution of early COVID-19 mortality in Mexico

Authors: Ramirez-Aldana et al.

General comments:
The researchers have an interesting dataset and applied a variety of spatial techniques to understand the distribution and state-level risk factors for COVID-19 in Mexico. Their manuscript has the potential to make an important contribution to our understanding of the disease, but I have the following major epidemiological concerns about their analytical approach:**

Thank you very much for the revision and for your time and effort in reviewing our paper. We have modified the paper attending to your comments and suggestions. A point-by-point response is provided here.

**1. Currently, most epidemiologists distinguish between risks (expressed as percentages) and rates (expressed as number of cases per person-years). While the authors state in some areas that the outcome variable is mortality rate, they express this outcome as a risk. There is also an issue with their denominator for measuring mortality risk/rate. The denominator should be the state population if they are measuring the mortality risk or state-level person-years for mortality rate. However, it appears that they are actually using the number of individuals who tested positive for COVID-19 (although this needs to be stated more clearly). As a consequence, they are modeling the case-fatality risk. This is a legitimate measure, but this needs to be clarified throughout the manuscript and issues associated with this epidemiological measure should be addressed. The mortality risk and rate are substantially lower than the case-fatality risk at this time, and the incorrect use of these terms will contribute to confusion in the literature.**

We are using the fatality risks associated with tested individuals (tested case-fatality risks or tCFRs). We are not using the state population as the denominator; first, because we believe that deaths are not representative of all real deaths in Mexico (see also **Answer 1 Reviewer 1**). The second reason is because we believe that if we used the total population as denominator, we should also use the population values associated with comorbidities, age, and other variables. In such case, we would only be using the information concerning deaths from the epidemiological data set, wasting all the valuable information included there. If we used the information from the epidemiological data set, and not from the population, with the denominator obtained from the population, we think that perhaps our analyses would be biased, since it is not appropriate to assume that the distribution of all variables, particularly health-related, in tested individuals is the same as in all the Mexican population. Thus, we are managing the data as it corresponded to a sample, though of course not representative, since it corresponds only to a very particular kind of individuals, those tested.

However; we also fitted models using as denominator the projected population by state in 2020 according to CONAPO (cited in the document) to see whether results were or not similar using the total state population (mortality risks) or tested individuals. Particularly, for our final model, the direction we found for the associations was similar using the projected population or tested individual numbers, though the estimated coefficients are not the same (shown below, the ones used for the manuscript are presented in **Answer 3 Reviewer 1**). When we obtained the quartile maps and spatial clustering associated with the risks using the projected population, the clusters and maps were very similar as the ones using the tested individuals, though obviously the values of the risks were much smaller. Below, we show the maps concerning spatial clustering. All these matters have been added to the discussion (**L-432 and L-450**).

Projected population (mortality risks)

```
Coefficients:
                      Estimate Std. Error z value Pr(>|z|)
(Intercept)         -12.589264   1.155213 -10.898  < 2e-16 ***
Diabetes             -0.003506   0.032561  -0.108  0.91426
Obesity               0.173593   0.019834   8.752  < 2e-16 ***
ICU (residuals)       0.211766   0.024618   8.602  < 2e-16 ***
GDP                   0.275804   0.041366   6.667 2.60e-11 ***
Internal migration    0.313695   0.035458   8.847  < 2e-16 ***
External migration    0.335123   0.128349   2.611  0.00903 **
Age group 10-39      -0.104250   0.018201  -5.728 1.02e-08 ***
Intubated (residuals) 0.261914   0.032008   8.183 2.77e-16 ***
Physicians            1.104937   0.071354  15.485  < 2e-16 ***
Cardiovascular       -0.106802   0.069668  -1.533  0.12527
Hospitalized (res.)  -0.088390   0.015863  -5.572 2.52e-08 ***
```

Projected population (mortality risks):

Tested individuals (paper):

**2. The researchers appear to have a great deal of high-quality patient-level data that they have aggregated for the purpose of fitting a state-level GGWR model. I would suggest that the authors consider fitting a mixed logistic regression model with a random intercept for state where they can examine associations with patient-level and contextual state-level variables. Age and sex are important for predicting case-fatality and the interpretation of these variables is subject to ecological bias when these individual level variables are averaged for what I assume are the COVID-19 positive patients in each state. In addition, based on the Moran’s I result, there does not appear to be strong justification to be concerned about spatial auto-correlation. It is also unclear if the GGWR model is appropriate for area data where spatial distances are measured between centroids of relatively large geographical areas.**

Since several patient-level analyses have already been performed for this data (References 4, 10 and 17 of the manuscript); whereas spatial analyses have not, we decided to use this approach. Additionally, at a state-level, we were able to add socio-economic and other type of variables not available in the epidemiological data set, e.g. GDP and mobility, not only using health-related variables, which are those mainly included in the epidemiological data.

However; we fitted for the disaggregated data, mixed logistic regression models with random intercept for state, and found more or less similar results as those with the aggregated data in terms of health variables. We first realized that in this case ICU, intubated, and hospitalized cannot be used in the same model, since all patients in ICU are hospitalized. Thus, we fitted two type of models, ones using intubated and ICU together, and others using hospitalized. Age was used as a continuous variable, but to be able to compare with our results, we decided to use the age-groups as in the revised version of our manuscript: 10-39, 40 to 69, and 70 and more. We also performed a correlation analysis, identifying that association between explanatory variables was in general lower than with the aggregated data, being the more important association that between variables hospitalized and pneumonia (positive), and between age groups of 40 to 69 and 70 years and over. Thus, we fitted models excluding these variables accordingly, whose results are shown below:

Including intubated and UCI

```
                      Value  Std.Error    DF    t-value p-value
(Intercept)        -3.771632 0.16356039 12287 -23.059567  0.0000
Age group 10-39    -1.221349 0.14017140 12287  -8.713253  0.0000
Age group 40-69     0.162950 0.09159918 12287   1.778943  0.0753
Diabetes            0.187471 0.08475436 12287   2.211936  0.0270
COPD                0.196479 0.13817722 12287   1.421936  0.1551
Inumno              0.110705 0.16448979 12287   0.673021  0.5009
Hypertension        0.203247 0.08452137 12287   2.404687  0.0162
CARDIOVASCULAR     -0.351672 0.15064348 12287  -2.334466  0.0196
Obesity             0.455093 0.08525372 12287   5.338102  0.0000
Kidney              0.056416 0.15288907 12287   0.369002  0.7121
PNEUMONIA           0.593247 0.09136229 12287   6.493348  0.0000
Smoking            -0.063336 0.12060045 12287  -0.525173  0.5995
ICU                 0.366239 0.11368431 12287   3.221548  0.0013
Intubated           1.170948 0.10887434 12287  10.755039  0.0000
```

Including hospitalized (pneumonia not included to avoid multicollinearity)

```
                       Value  Std.Error    DF   t-value p-value
(Intercept)        -5.959923 0.16686272 51316 -35.71752  0.0000
Age group 10-39    -1.477627 0.11490139 51316 -12.85996  0.0000
Age group 40-69     0.005564 0.07339710 51316   0.07581  0.9396
Diabetes            0.322264 0.06809666 51316   4.73245  0.0000
COPD                0.096127 0.11300293 51316   0.85066  0.3950
Inmuno              0.118753 0.13565292 51316   0.87542  0.3814
Hypertension        0.261577 0.06790968 51316   3.85184  0.0001
CARDIOVASCULAR     -0.289284 0.12216176 51316  -2.36804  0.0179
Obesity             0.548126 0.06757241 51316   8.11168  0.0000
Kidney              0.059414 0.12457103 51316   0.47695  0.6334
Smoking            -0.069364 0.09800288 51316  -0.70778  0.4791
Hospitalized        2.846097 0.09713465 51316  29.30053  0.0000
```

What we observed is that apart from the confounders (hospitalized and intubated), the variables with greatest significant association in both models were diabetes, obesity, age group of 10 to 39, as in our models, plus hypertension and cardiovascular, which were not significant in our model. However; the later variable has a negative association, which we found in some of the models we fitted, but not consistently. Thus, in our model we were able to find similar effects (direction) than at an individual level, plus identifying the effects of other variables not available at an individual level. All these matters have been added to the Discussion (**L-443**).

Age was analyzed once again to obtain more adequate age groups as explained above in **Answer 9 Reviewer 1**.

It is true that global Moran’s I was not significant, but the local analyses indicate significant spatial clustering; and thus, we think this justifies our approach. Additionally, GGWR are not based on the spatial weight matrix used for the Moran’s I, as other models, for instance spatial-lag or spatial-error models, only on distances, as correctly stated by the reviewer. The affirmation that distances might be relatively large is a concern we shared with the reviewer; though others have performed similar analyses with larger distances, even at a country level. It was not possible to apply spatial analysis to a lower aggregation level, municipalities, since at this stage of the disease, there was a large presence of zero values. This makes impossible to apply common spatial techniques, since as far as the authors know, there are not zero-inflated techniques analogous to GGWR, spatial-lag models, etc. However; we changed the kernel associated with the GGWR from a fixed to an adaptive one to better consider the difference of distances in all the country (**L- 196**).

**3. The authors’ model fitting process does not appear to consider issues like confounding and interaction effects. This should be addressed in their methods and results.**

As explained in **Answer 5 Reviewer 1**, the selection process was modified. In the final model obtained for this revised version, we experimented using interaction effects, but they were not significant (**L-288**). We now define clearly which variables are confounders. We considered ICU, intubated, and hospitalization as confounders. Hence, we want to understand the association between comorbidities, age, socio-demographic variables, economic variables, temperature, and variables associated with mobility with the risk of death controlled for the confounders (**L-145**).

**4. The authors may want to consider using Kulldorff’s spatial scan statistic or Tango and Takahashi’s flexible scan statistic (both available in R and a freeware) for high and or low cluster detection especially if they want to consider clusters of varying sizes and/or shapes.**

Thank you very much for the suggestion. We used the Kulldorff’s spatial scan statistic in our data and obtained a cluster in the Northwest including the Baja California Peninsula, which makes sense since it includes areas in which there were higher fatality risks and corresponds to the frontier. Interestingly, when we used the projected number of individuals by state in 2020 as denominator (mortality risks), a High-High cluster appeared also in the Baja California peninsula.

As far as we understand, Kulldorff’s statistic, and associated clustering, is based on spatial point processes associated with each area (centroids) and distributions associated with counts (Poisson distribution) and/or phenomena with success (disease) and failure (Bernoulli distribution). The analysis includes as null hypothesis that the probability of success in a zone is similar as the probability of success outside. The likelihood ratio is used as statistic and the statistic can be used for inference and to identify clustering, cluster of zones in which the null hypothesis was rejected. Then, Monte Carlo simulations are used since the distribution of the statistic is unknown. On the other hand, the clustering we obtained is based on contiguity-based spatial weights (as Moran’s I) and local inference (local indicators of spatial association, LISA), after that inference is also based on simulations. Thus, as we see it, both methods seem to be complimentary, and yet different, ways of analyzing clustering, both with advantages and disadvantages, being the LISA methodology closer to the Moran’s I methodology, which perhaps is the most known measure of spatial autocorrelation. Then, to be able to have a more direct comparison and accordance with the Moran’s I presented in our paper, we decided to let this analysis in the main text, but we discuss the results obtained with this other type of clustering (**L-436**).

Specific comments:
**Please note that based on the above major concerns, I will only provide specific comments that might assist in revising the manuscript.

i. Please edit the title so it is clear that case-fatality risk is being explored or make appropriate changes to the dependent variables in all the analyses.**

The title was modified to reflect the type of risks that are being analyzed. However, as mentioned above (**Answer 1 Reviewer 2**), we used the projected values as denominator for the risks (mortality risks) in different analyses (spatial distribution and clustering, and spatial modelling) and made comparisons with analyses using the tested number of individuals. The justification of why the latter were used was included in the manuscript (**L-450**).

**ii. Line 29. “Torpid” means inactive or lethargic. I don’t believe this is what the authors mean to say.**

Thank you, we have removed and modified this term.

**iii. Please make certain footnotes for all tables and figure legends are in English or include English translations. Please upload figures of higher resolution. They were very difficult to read. Please note on Table 1 that “pneumonia” is misspelled in the column heading and make certain any short forms have appropriate footnotes (e.g., “Age_d”). In addition, footnotes might be needed for several column headings. For example, it is unclear what the "Number of residents" represents. Is it the population in units of 1000, the number of residents infected with the virus, or the number tested?**

All these problems were corrected in all figures and tables.

i**v. Please use the term “multivariable” rather than “multivariate” to indicate a model with multiple independent variables. The current recommended convention is to use the term multivariate for statistics that have more than one dependent variable.**

This problem was corrected in all the manuscript, univariable was used as well instead of univariate.

**v. Make certain in the methods that it is stated how the linearity assumption between the independent variable and the outcome was assessed for any models fitted, and clearly state which diagnostics were performed for all models fitted.**

Thank you very much for this suggestion, though we had fully covered other aspects, we are now being more careful with the linearity assumption. We evaluated linearity by obtaining scatter plots between each variable and the log-transformed risk (as used in the model) with the help of a smoothed LOESS (locally estimated scatterplot smoothing) curve (an instance is shown below for age) and trying to apply different transformations in some variables in which linearity could be improved **(L-209)**. In particular, GDP was transformed obtaining better linearity with the response. We performed and now added to the manuscript the goodness-of-fit evaluation and model diagnostics (**L-223, L-281**). Additionally, in the discussion we add some lines concerning goodness-of-fit and model assumptions (**L-425**), including the fit of a quasi-Poisson model.

**vi. Please clarify on line 189 that “number of people tested with COVID-19” indicates the number of people that tested positive for COVID-19.**

As explained before in **Answer 1 Reviewer 2** and **Answer i Reviewer 2** it is correct the use of “number of people tested”.

**vii. In line 203, the authors should state “****The significance level for all analyses was 5% (i.e., alpha=0.05)”.**

Thank you, we have modified this sentence as suggested.

**viii. In line 215, the authors state they used a “Bayes spatial technique”. Do the authors mean they performed the clustered analyses on data smoothed using a Bayesian technique or some other Bayesian process to estimate the p-values? This should be clarified in the methods, and I would strongly recommend against performing cluster-detection methods on smoothed data since the smoothing process can create clusters.**

The Bayesian technique was only used to obtain smoothed risks, and it is only used to consider that small populations have large variance and vice versa, which occurs since the denominator in the variance formula depends on the population size **(L-167)**. We performed analyses with both the raw and smoothed values obtaining in the case of the quartiles, similar maps, but not the same, as was already had stated since the previous version (**L-239**), and with exactly the same cluster and significance maps (**L-249**). We now clearly state that analyses, both for the risks distribution and spatial clustering, were performed with both raw and smoothed values **(L-169)**. We identified some errors in the values used before in the discussion for the risks and corrected them (**L-383**).

**ix. Figure 1. Please note that relative risk and excess risk are not synonyms. Excess risk is a synonym for risk difference.**

Luc Anselin (creator of Geoda) uses both terms indistinctly, though in a spatial sense. For instance for a country, he defines it as the quotient between the number of events and the expected number of events, considering for the latter, as if the risk associated with a state were the same as the national risk. We think this definition is closer to a relative risk, though perhaps not exactly the same, so that now we have added more clarification of what we are talking about in the manuscript (**L-171**).

**x. Line 236. The authors should explain that VIF was initially used to assess multicollinearity in the methods. In the methods, they should state what threshold was subsequently used to determine if two independent variables were highly correlated. I would suggest assessing the univariable statistical significance of each variable as part of the process of considering variables for inclusion in the multivariable model.**

We have added that VIF was used to assess multicollinearity (**L-210**). The suggestion to considerate univariable analyses was very valuable, thank you. The selection process was modified considering this aspect as explained above (**Answer 5 Reviewer 1**), and the correlation measures are not used in the selection process, only as descriptive measures we still include in the paper, adding all the variables we calculated for this revised version (**Figure 3** and **L-271**) and the raw risks, instead of corrected risks, following **Answer viii Reviewer 2** and that the raw values are the ones used in the models. The cut-off value of VIF used to determine multicollinearity presence was 10.

**xi. Please make sure to state the measure of association estimated from the exponentiated coefficients and present them in table form.**

The multiplicative effects over the tested case-fatality risks (exponentiated coefficients) are presented now instead of the coefficients in **Table 2.**

**xii. Lines 278-308. Avoid the use of the phrases “x% increase” or “x% decrease” when describing measures of associations (e.g., incident rate ratios, relative risks, or odds ratios). They are ratio measures and the value after subtracting from 1 (e.g., 1.73-1) can vary depending on the referent category being used for categorical variables or the direction of change for continuous variables. For instance, if the odds ratio for sex was 4 with females as the referent, would the authors be comfortable stating that the odds were 300% greater (i.e., 4-1/1=3) for males than females, but if the referent were males, making the OR= 0.25 (i.e., OR=1/4), state the risk was decreased 75% for females (0.25-1/1)? I would recommend simply stating if the odds, risk, or rate was significantly higher or lower for one category vs. another, or stating whether there was a significant positive or negative association for linear continuous variables. The authors can refer to a table for specific values or include the measure of association, its 95% CI, and p-value in brackets beside the statement.**

All results were modified as suggested by the Reviewer (**From L-325**)
