# Supplementary material for: Spatial epidemiological study of the distribution, clustering, and risk factors associated with early COVID-19 mortality in Mexico
Source: PLoS One. 2021 Jul 21;16(7):e0254884. doi: 10.1371/journal.pone.0254884 (PMC8294534; doi:10.1371/journal.pone.0254884)
Supplement: Attachment — Submitted filename: Answer_reviewers_24022021.docx [file pone.0254884.s002.docx]

February 20^th^, 2021

**Dr Agricola Odoi**

**Academic Editor**

**PLOS ONE**

It is a pleasure to submit, on behalf of all authors, the reviewed version of our manuscript number PONE-D-20-26970R1, entitled “**Spatial epidemiological study of the distribution, clustering, and risk factors associated with early COVID-19 mortality in Mexico**” by Ricardo Ramírez-Aldana, Juan Carlos Gomez-Verjan, Omar Yaxmehen Bello-Chavolla and Carmen García-Peña.

We thank the reviewers for their valuable feedback, commentaries, and suggestions on our manuscript, which we have found very helpful and constructive. Accordingly, we have modified the manuscript following each of the reviewer's commentaries and suggestions. We hope our manuscript's reviewed version is more suitable and complete for eventual publication in your prestigious journal. Please find below our responses to the reviewer's commentaries and suggestions.

We look forward to hearing from you at your earliest convenience.

Sincerely yours,

Dr Ricardo Ramírez-Aldana,

Medical Researcher,

Research Division,

Instituto Nacional de Geriatría (INGER)

Periférico Sur No. 2767

Col. San Jerónimo Lídice,

Del. La Magdalena Contreras, D.F. 10200

ricardoramirezaldana@gmail.com

**Reviewers' comments:**

**Reviewer's Responses to Questions**

**Comments to the Author**

**1. If the authors have adequately addressed your comments raised in a previous round of review and you feel that this manuscript is now acceptable for publication, you may indicate that here to bypass the “Comments to the Author” section, enter your conflict of interest statement in the “Confidential to Editor” section, and submit your "Accept" recommendation.**

Reviewer #1: All comments have been addressed

Reviewer #2: (No Response)

**2. Is the manuscript technically sound, and do the data support the conclusions?**

**The manuscript must describe a technically sound piece of scientific research with data that supports the conclusions. Experiments must have been conducted rigorously, with appropriate controls, replication, and sample sizes. The conclusions must be drawn appropriately based on the data presented.**

Reviewer #1: Yes

Reviewer #2: Partly

**3. Has the statistical analysis been performed appropriately and rigorously?**

Reviewer #1: Yes

Reviewer #2: Yes

**4. Have the authors made all data underlying the findings in their manuscript fully available?**

**The** [PLOS Data policy](http://www.plosone.org/static/policies.action#sharing) **requires authors to make all data underlying the findings described in their manuscript fully available without restriction, with rare exception (please refer to the Data Availability Statement in the manuscript PDF file). The data should be provided as part of the manuscript or its supporting information or deposited to a public repository. For example, in addition to summary statistics, the data points behind means, medians and variance measures should be available. If there are restrictions on publicly sharing data—e.g. participant privacy or use of data from a third party—those must be specified.**

Reviewer #1: Yes

Reviewer #2: Yes

**5. Is the manuscript presented in an intelligible fashion and written in standard English?**

**PLOS ONE does not copyedit accepted manuscripts, so the language in submitted articles must be clear, correct, and unambiguous. Any typographical or grammatical errors should be corrected at revision, so please note any specific errors here.**

Reviewer #1: No

Reviewer #2: Yes

**6. Review Comments to the Author**

**Please use the space provided to explain your answers to the questions above. You may also include additional comments for the author, including concerns about dual publication, research ethics, or publication ethics. (Please upload your review as an attachment if it exceeds 20,000 characters)**

**Reviewer #1: This paper examines the factors associated with early COVID-19 tested case fatality in Mexico at the state level. The authors find that both internal and external migration are important, as are health risk factors (counts of diabetes, hypertension, and ICU hospitalization). Physician density also has a strong positive association.**

**The authors have satisfactorily addressed all of the issues that I and the other reviewer raised. I'm impressed, both because together we had a very large number of comments, and because of the excellent, detailed responses. The resulting paper and its focus on t-CFRs strikes me as being a significant improvement. The limitations that remain either will be addressed in parallel or subsequent work (for example, using individual rather than grouped observations) or are inherent and cannot be fully addressed (specifically, the highly selected nature of early tests).**

Thank you very much for your revision and comments. We appreciate your time in reviewing and commenting on our paper. We have modified the paper attending to all your suggestions. A point-by-point response to every raised issue is provided below.

**My remaining comments are secondary:**

**1. Most important of all, the paper needs to be reviewer by a native writer of English. Every page has awkward phrasings, though there are few if any grammatical errors. The last paragraph in the paper (lines 432-478) needs to be broken up into several pieces.**

The English was carefully reviewed to avoid grammatical errors and incorrect phrasing. According to **Answer 7 Reviewer 2**, lines 432-478 have been eliminated and one part was simplified, please refer to the manuscript.

**2. Line 72: note that OCDE is abbreviated as OECD in English**

Thank you for the observation; we have corrected this mistake.

**3. Line 76: note that population aging makes the pyramid become more rectangular rather than flattened.**

Thanks for the observation; we have corrected this error.

**4. Line 223: “the corresponding estimated parameters do not have a meaning” sounds jarring and needs to be restated. Indeed, while I am happy with the use of residuals from prior regressions, the rationale for doing so should be explained. This leads to a bigger issue for the authors and the editor: the paper lays out much technique without explaining why various approaches are being taken. This is fine if the audience consists of epidemiologists (or quantitative demographers or econometricians), but if the readership is more general, they are likely to treat the techniques as a black box. Of course, lots of detail adds to length, which is also costly.**

Thanks for the observation; we have rewritten what was contained in line 223 (previous version) to make it clearer (lines 234-243), eliminating the part in which we stated that parameters have no meaning.

We have tried to explain in the methods section why each analysis was performed in simpler sentences in those cases in which this was not included before. Please refer to the main manuscript in the methods section.

**5. It is important (around line 400) to be careful about the use of causal language. For prior comorbidities (diabetes, obesity), this makes sense. It is less clear in the case of migration (as migratory links might lead people to travel for health care) but still fairly plausible. It is implausible in the case of variables like physician density of GDP/capita since diagnosis and testing will be different.**

Thanks for your commentary. We agree that chronic conditions are more clearly connected with COVID-19. Regarding migration, our explanation is more related to infectious diseases incidence due to people transit between towns or cities. It is true that other variables such as physician ratio or GDP could be seen as implausible, so we have corrected the redaction on the discussion, and now it is clearer in terms of the type of relationships we found and the reasons behind these findings. Additionally, we eliminated GDP from the sentence in which it was included with a causal connotation and modified other lines accordingly, so it does not read as an association. Please refer to the main text.

Reviewer #2: Manuscript #: PONE-D-20-26970-R1

**Title: Modelling the impact of health-related variables, age, migration, and socio-economic factors in the geographical distribution of early tested case-fatality risks associated with COVID-19 in Mexico**

**Authors: Ramirez-Aldana et al.**

**General comments:**

**The authors have done a very thorough job responding to reviewers' questions and suggestions.**

Thank you very much for your time and effort in reviewing our paper. We have modified the paper attending to all reviewer’s comments and suggestions. A point-by-point response is provided here.

**However, the following issues need to be addressed in the revised manuscript:**

**1. The authors spend part of the discussion writing about variables measured at different hierarchical levels. In their response letter to the first review, they do not seem to recognize that multi-level models not only include random intercepts at various hierarchical levels but also allow for the proper estimation of variables measured at these different levels (e.g., individual person, municipality, and state). One of the major limitations of their modelling technique (geographic weighted regression) for their study is that it required the authors to take individual level variables and make them ecological variables (e.g., age of the individual is now the proportion of the sample in an age category). Consequently, all their results are subject to ecological bias (i.e., the ecological fallacy). Similarly, the ability to detect interaction effects is reduced in ecological studies. However, if the authors revise the discussion to include issues related to ecological bias, I think that would be sufficient. It is unfortunate that the GWR approach was not more revealing. I suspect most if not all of the variation in variable coefficients attributed to differences among states would have fallen within the 95% CI of the estimates from the global model.**

**The authors may wish to review material from the Centre for Multi-level Modeling (**[**http://www.bristol.ac.uk/cmm/learning/multilevel-models/what-why.html**](http://www.bristol.ac.uk/cmm/learning/multilevel-models/what-why.html)**) and other references where multi-level models have been used for epidemiological studies. However, based on their objectives, I do not expect the authors to change their modeling approach.**

Thank you very much for your detailed response. We are aware that variables in a generalized mixed linear model can belong to different aggregation levels considering the nesting between observations. However, our idea was to analyze the aggregated data to have a better understanding of the spatial distribution of the mortality and variables affecting it at that level, representing mortality through maps. Additionally, in terms of the model, we think that possible spatial autocorrelation not always can be solved by only considering the nesting effects in a generalized linear mixed model (GLMM). Of course, the correlation structure in the GLMM in terms of the error could have been considered, but this could overcomplicate the model. In the previous review, we understood that one of the reviewers was pointing out that the variables at an individual level could have a different association with the risks in an aggregated compared with a disaggregated model. We are now aware that what the reviewer intends is for us to acknowledge in the manuscript, the fact that aggregating the data has some drawbacks, particularly when trying to infer the results to an individual level. We have added to the discussion a part (lines 469-471) about the limitation of our study in terms of an ecological fallacy at the time of the interpretation of the data.

**2. If the authors are committed to presenting their GWR model, they should include the results of the test for bandwidth or other tests to support that the GWR model fits better than the “global model”. In addition, the authors should present the tests of non-stationarity for each variable. Only if there’s evidence of non-stationarity should the authors present results suggesting that there are different effects among states for a particular variable.**

As far as we know, there is no a bandwidth test or a test comparing a generalized geographically weighted regressions (GGWR) with the global model as in a geographically weighted regression (GWR). In GWR, this type of tests is based on the squared sum of residuals (SSR) associated with the model fitted by ordinary least squares (OLS) and the GWR model. These tests are based on a statistic with an associated F distribution whose degrees of freedom have been calculated. Thus, we decided to present the SSR corresponding to the GGWR and its equivalent generalized linear model (GLM), as well as the values of the $R^{2}$ associated with each model and the Akaike Information Criterion (AIC) (lines 250-252). We explored using the tests corresponding to the GWR equivalent with our GGWR, i.e. assuming normality; however, we discarded them since these results could be unreliable, considering that a different distribution is used. We have added the mentioned statistics in the manuscript (lines 316-318). We have tested whether the spatial correlation is significant for each of the inputs in the models, determining that in some cases, there is significant autocorrelation (lines 303-306). To measure spatial autocorrelation for these variables, we used the Moran’s I statistic with queen contiguity weights and corresponding tests to be consistent with the rest of the manuscript.

**3. In terms of their risk maps, the authors should strongly consider adjusting for sex and age (e.g., indirect standardization). Currently, the maps may only be showing demographic differences among the people tested within these states. Similarly, the LISA statistics should adjust for age and sex. I believe this can be done with LISA and it certainly can be done with the spatial scan statistic.**

We have adjusted the risks for sex and age using a standardization, in which, based on Preston et al. (2000), we used as a standard the average of the 32 states for age and sex compositions. For the standardization process, in terms of age, we used the same age groups derived from the models. We updated the maps in Figure 1 accordingly. We applied a similar standardization process for the LISA and spatial autocorrelation maps in Figure 2. We have added this process to the methodology section (lines 169-171) and modified the results and discussion according to the new obtained values.

**4. In terms of terminology, technically an observed/expected ratio is referred to as a standardized morbidity/mortality ratio (SMR) rather than a relative risk. Please note that using indirect standardization, an SMR can be estimated that accounts for age and sex distribution in estimating the expected count.**

Thank you, we have realized that effectively the name used in epidemiology for the ratio we used is standardized mortality ratio (SMR). Thus, we have changed the name in all the document, and performed the corresponding indirect standardization process, calculating for the denominator the expected values in each state considering the national structure according to age and sex. For the numerator, we obtained the raw observed values considering the age and sex structure. The obtained quotients were similar to those obtained with the process without adjusting for age and sex. According to these new values, we modified the corresponding map in Figure 1, as well as the values presented in the Results and Discussion. Additionally, we added in Methods this process as part of the analyses (lines 175-177).

**5. Based on the authors’ response, it is now clear they are modelling the risk of mortality from COVID -19 among all tested individuals not just those that tested positive for COVID-19. Consequently, it would be clearer if they used the phrase** **mortality risk from COVID-19 among tested individuals and use “MRt” as an acronym and avoid the current “t-CFR” since a case-fatality risk refers to the risk of mortality among those who have the disease not those tested for the disease. Even with the definition provided in the current text, altering the meaning of a commonly used epidemiological term will confuse many readers.**

We have modified and replaced the term “t-CFR” for “MRt” in all the manuscript and used mortality risk from COVID-19 among tested individuals for the definition and other parts of the document in which the other term, tested case-fatality risks, was used.

**6. In the discussion, the authors should avoid commenting on aspects of COVID-19 that are not examined in their study. For instance, risk factors for death may not be the same as those for the disease, so make certain associations are explained in this context. Similarly, avoid comments about the propagation of disease (e.g., lines 357-359) when space-time clusters were not examined nor were space-time factors considered in the GWR models.**

We have modified the discussion, so now we focus on mortality risks and state that information corresponds to the early stages of the outbreak. Please refer to the main text (lines 386-388).

**7. The authors have included descriptions in the discussion of a large number of analyses that are not presented in the methods or results. While these comments were appropriate for their response letter, they should not be included in the current manuscript unless the authors wish to include a comparative methodology component to the manuscript. However, if they choose to add this element, I would encourage them to split the manuscript in two and focus on spatial mapping and cluster detection in one manuscript and the risk factor analyses in another. In each paper, they could include all the methods being considered in the methods section and provide the accompanying results.**

We have eliminated the description of the analyses we performed for the previous response letter, only leaving those lines corresponding to the robustness of our methodology or parts relevant for the discussion, but without including any details of the analyses.

**8. There is some confusion about the nature of hospitalization, intubation, and ICU use in the authors’ analyses. At the individual-level, these are intervening variables and not confounding variables. Typically, intervening variables are not included in a model and confounders are usually controlled for by their inclusion in the model. The authors might want to clearly provide a definition of a confounding variable and clarify if they are using the epidemiological definition.**

Thanks for your observation; we used a model in which the confounding variables are not interpreted but are included since they control the final effect as in Econometrics or experimental design analyses. We think that hospitalization, intubation, and ICU at an aggregated level can be considered as a proxy of how the severity of the cases is geographically distributed, which certainly is associated with the mortality risks by state. This severity among other things could depend over several factors, for instance whether individuals waited too much to attend hospitals, and of course on morbidities. If they were omitted from the model, they would form part of the error term, and we think that the severity of the disease represented by these variables should be included to avoid spurious effects in terms of the variables we are interested in. Thus, we are controlling for the severity of the disease by state by including all these variables, in a previous revision, we explained that using the variables as they are, as principal components, or as residuals of models to consider and eliminate the effects other inputs have over them (as they are presented in the paper) gives similar results. Additionally, in a previous revision, we were asked whether an interpretation of such variables was necessary since we had included it before and eliminated it afterwards. We have included these aspects in the methods section.

**9. The authors should be cautious throughout the abstract, results, and discussion about the use of the term “impact”. Measures of effect (e.g., attributable fraction, population-attributable fraction) are often used to measure the impact of controlling for a variable; a variable may have a large relative risk, but its impact on the population may be quite small since the exposure is rare. I would recommend simply commenting on the direction and statistical significance of the measures of association in the text and not ranking the “impact of these variables”.**

Thanks for your suggestion; we have avoided the use of the term “impact” throughout all the manuscript.

**10. Clarify if you are using a Poisson model to estimate incident rate ratios or relative risks in the methods and in the results.**

We did not use a Poisson model in these calculations. The risks now are adjusted for sex and age, as requested by the reviewer.

Specific comments:

Title:

**i. Perhaps a better title would be** **“Spatial epidemiological study of the distribution, clustering, and risk factors associated with COVID-19 mortality in Mexico”.**

Thank you very much; we modified the title of the manuscript to include what was proposed plus the term “early” to distinguish the time in which the data were collected.

**Introduction:**

**i. Line 58. I do not believe “Spanish Influenza” needs to be in italics.**

We corrected this term.

**ii. Line 72. It should read “according to the OCDE” and “OCDE” should be written in full the first time the acronym is introduced.**

We corrected this part.

**iii. Line 77. It would be better to state, “****are likely to lead to higher rates of mortality”.**

We corrected this line.

**iv. Line 92. Replace “variate” with “vary”.**

We modified this term.

**v. Lines 100-101. It should read, “…****in Mexico that should be examined in more detail to better understand…”.**

We modified this part as requested.

**vi. Line 105. Replace “A first set of variables” with “Individual level variables” and then edit the rest of the sentence to reflect this change.**

We modified this sentence accordingly.

**vii. Line 109-110. It should read “****who were suspected of having COVID-19”.** **In a following sentence or within this sentence clarify if testing required suspicion of disease by a physician and/or if members could receive a test based on their own concerns.**

Thanks for your commentary; we have corrected this line.

**viii. Lines 115-116. Make it clear here and throughout the manuscript that you are looking at the** **proportion of individuals in these age groups.**

We corrected this term here and in other parts of the manuscript. In these lines, the structure of the manuscript corresponds to present the variables first and then explaining the aggregation process later, so this was considered for the correction. However, when variables are mentioned in general by a simple name, we let the name as it was.

**ix. Line 121. Please replace “rate” with “risk”.**

Replaced

**x. Line 126. Please replace “clustering” with “****population density in various contexts” to avoid confusion with clustering in the statistical sense.**

We modified this part; however, to avoid the use of the term population density twice in a row, we applied a slight modification to the following part in the sentence.

**xi. Lines 133-134. You should avoid dropping data to achieve linearity. If necessary, model the polynomial or categorize the variable (i.e., classify the state GDP into quantiles).**

We did not drop any data; we calculated a variable that corresponds to the interaction (multiplicative effect) between a dummy variable concerning states with the largest cities in Mexico and GDP; thus, this variable has a value of zero in those states. Other transformations, including the calculation of quantiles, did not work since these cities can be considered as outliers. We tried to be less technical in the description of the process here, but we have modified this sentence since it could be confusing.

**Results:**

**i. Lines 119-123. The text is very unclear for this section. Please see my comments (general comments section) concerning these variables being intervening variables and edit accordingly or justify the approach used to control for the effect of these variables.**

Thanks for your commentary; we have already solved this issue.

We think the reviewer refers here to the use of the residuals and the confounders. We have modified the Methods section according to **Answer 4 Reviewer 1** to better explain this.

**ii. Lines 281-289. Please note that for Poisson models there is no assumption of homogeneity of variance or normality. However, Anscombe residuals will have a normal distribution if the model fits the data. In addition, the null-hypothesis for the overall model p-value is that all coefficients equal zero and is not intended to test for lack of model fit.**

We are aware that there is not a normality assumption in generalized linear models (GLM); however, it has been shown that standardized Pearson residuals (e.g. Agresti, 2002, p. 367) have an asymptotic normal distribution in Poisson and loglinear models, and we effectively referred here to the model fit when the normal distribution was mentioned. We also forgot to include Pearson in the name of the residuals, but now have added it. We are also aware that homogeneity of variance is not an assumption in GLM (though a weight term associated with the dispersion parameter can be included); in fact, since overdispersion can be present considering that the mean and variance are related under a Poisson distribution, we included a measure of overdispersion (Phi). Effectively, the type of hypothesis we performed does not correspond to the use of the residual deviance, but to what is called an omnibus-type test, a likelihood ratio test (LRT) in which it is compared the model with only the constant term with a model with all the parameters, seeing whether all coefficients are jointly zero. Thus, we modified this sentence accordingly.

**iii. Throughout the figures and tables concerning your Poisson models, include a footnote that the** **offset is the log of the total number of people tested in the state and indicate what measure of association is being estimated.**

We have included footnotes, when appropriate, corresponding to the offset and measure; the name of this measure was taken from Rothman K, Grenland S, Lash T. (2008). Modern Epidemiology. 3a ed Wolters Kluwer, p. 610. Additionally, the figures and tables included titles that more clearly represent the analyzed measure, the mortality risk from COVID-19 among tested individuals.

**iv. Table 2. Include the 95% CI for the global effect for each variable.**

We have included the confidence interval in Table 2.

**v. Lines 313-314. Make certain it is clear that variables measured at the individual level have been collapsed by writing “diabetes (%)” rather than just “diabetes”, for example.**

We have modified all the document to include this suggestion.

**vi. Line 350. Are there any diagnostics for GWR models? If so, include them here and describe them in the methods.**

As we have discussed in **Answer 2 Reviewer 2**, as far as the authors know, there are diagnostics for GWR models, but not for GGWR. Thus, we have only included the squared sum of residuals (SSR), the values of the $R^{2}$, and the Akaike Information Criterion (AIC). This was included in the methods and results sections.

**Discussion: Please edit based on the suggestions in my general comments.**

We have modified this part according to all the suggestions of both reviewers.

**References: Please make sure article titles are formatted consistently.**

We have checked the consistency of the references and added some references.

**Figures: Make sure that the same symbols are used for ranges in categories for all figures and include a footnote that the** **brackets beside these categories concern the number of states in each category.**

Figure 1 included different symbols for the ranges (without parenthesis in one); it now corresponds to SMR adjusted for sex and age and includes intervals. We have added the footnotes in Figure 1 and 2, which include the meaning of the quantities in brackets.

**7. PLOS authors have the option to publish the peer review history of their article (**[**what does this mean?**](https://journals.plos.org/plosone/s/editorial-and-peer-review-process#loc-peer-review-history)**). If published, this will include your full peer review and any attached files.**

**If you choose "no", your identity will remain anonymous, but your review may still be made public.**

**Do you want your identity to be public for this peer review?**

Reviewer #1: No

Reviewer #2: No
