# Supplementary material for: Spatial epidemiological study of the distribution, clustering, and risk factors associated with early COVID-19 mortality in Mexico
Source: PLoS One. 2021 Jul 21;16(7):e0254884. doi: 10.1371/journal.pone.0254884 (PMC8294534; doi:10.1371/journal.pone.0254884)
Supplement: Attachment — Submitted filename: Answer_reviewers_10052021.docx [file pone.0254884.s003.docx]

May 10^th^, 2021

**Dr Agricola Odoi**

**Academic Editor**

**PLOS ONE**

It is a pleasure to submit, on behalf of all authors, the reviewed version of our manuscript entitled “**Spatial epidemiological study of the distribution, clustering, and risk factors associated with early COVID-19 mortality in Mexico**” by Ricardo Ramírez-Aldana, Juan Carlos Gomez-Verjan, Omar Yaxmehen Bello-Chavolla and Carmen García-Peña.

We thank the reviewers for their valuable feedback, commentaries, and suggestions on our manuscript, which we have found very helpful and constructive. Accordingly, we have modified the manuscript following each of the reviewer's commentaries and suggestions. We hope our manuscript's reviewed version is more suitable and complete for eventual publication in your prestigious journal. Please find below our responses to the reviewer's commentaries and suggestions.

We look forward to hearing from you at your earliest convenience.

Sincerely yours,

Dr Ricardo Ramírez-Aldana,

Medical Researcher,

Research Division,

Instituto Nacional de Geriatría (INGER)

Periférico Sur No. 2767

Col. San Jerónimo Lídice,

Del. La Magdalena Contreras, D.F. 10200

ricardoramirezaldana@gmail.com

Journal Requirements:

**Reviewers' comments:

Reviewer's Responses to Questions**

**Comments to the Author**

**1. If the authors have adequately addressed your comments raised in a previous round of review and you feel that this manuscript is now acceptable for publication, you may indicate that here to bypass the “Comments to the Author” section, enter your conflict of interest statement in the “Confidential to Editor” section, and submit your "Accept" recommendation.**

Reviewer #2: (No Response)

**2. Is the manuscript technically sound, and do the data support the conclusions?

The manuscript must describe a technically sound piece of scientific research with data that supports the conclusions. Experiments must have been conducted rigorously, with appropriate controls, replication, and sample sizes. The conclusions must be drawn appropriately based on the data presented.**

Reviewer #2: Yes

**3. Has the statistical analysis been performed appropriately and rigorously?**

Reviewer #2: Yes

**4. Have the authors made all data underlying the findings in their manuscript fully available?

The** [**PLOS Data policy**](http://www.plosone.org/static/policies.action#sharing) **requires authors to make all data underlying the findings described in their manuscript fully available without restriction, with rare exception (please refer to the Data Availability Statement in the manuscript PDF file). The data should be provided as part of the manuscript or its supporting information, or deposited to a public repository. For example, in addition to summary statistics, the data points behind means, medians and variance measures should be available. If there are restrictions on publicly sharing data—e.g. participant privacy or use of data from a third party—those must be specified.**

Reviewer #2: Yes

**5. Is the manuscript presented in an intelligible fashion and written in standard English?

PLOS ONE does not copyedit accepted manuscripts, so the language in submitted articles must be clear, correct, and unambiguous. Any typographical or grammatical errors should be corrected at revision, so please note any specific errors here.**

Reviewer #2: Yes

**6. Review Comments to the Author

Please use the space provided to explain your answers to the questions above. You may also include additional comments for the author, including concerns about dual publication, research ethics, or publication ethics. (Please upload your review as an attachment if it exceeds 20,000 characters)**

**Reviewer #2: Manuscript #: PONE-D-20-26970R2
Manuscript title: Spatial epidemiological study of the distribution, clustering, and risk factors associated with early COVID-19 mortality in Mexico
Authors: Ramirez-Aldana et al.**

**General comments:**

**The authors have done a thorough and thoughtful job in addressing reviewers’ questions and concerns in the revised manuscript and response letter. Below I have included editorial suggestions to make the text clearer and have some requests for clarification. In the future the authors need to have their work properly edited.**

Thank you very much for your revision and comments. We appreciate your time in reviewing and commenting on our paper. We have modified the paper attending to all your suggestions, which have greatly improved the manuscript. A point-by-point response to every raised issue is provided below.

**Specific comments:**

**1. Line 33: It should read, “Among health related variables,……”**

Thank you, this line was corrected.

**2. Lines 35-36: It should read, “external and internal migration had positive associations……..mortality risk with external migration having the second-highest positive association. As an indirect measure of urbanicity, population density…………………………overcrowding in households……”
Note: “Urbanicity” means the degree to which a given geographical area is urban while “urbanity” also refers to being refined.**

Thank you, this line was corrected and the term urbanicity is used instead of urbanity throughout the manuscript.

**3. Line 40: It should read, “10 to 39 years had a negative association with MRt.”**

Thank you, this line was corrected.

**4. Line 44: It should read, “fatality risk varied by zone.”**

Thank you, this line was corrected.

**5. Line 60: It should read, “in nursing homes.”**

Thank you, this line was corrected.

**6. Line 71: Please remove “On the other hand”. It does not properly link the two sentences.**

Thank you, this adverb was eliminated from the phrase.

**7. Line 73: It should read, “conditions that are now linked to…”**

Thank you, this line was corrected.

**8. Line 84: It should read, “increasing….”**

Thank you, this word was corrected.

**9. Lines 90-92: It should read, “are distributed throughout a territory, detect spatial clusters, and measure how the effects of variables associated with this risk vary within any given territory.”**

Thank you, this line was corrected.

**10. Line 92: It should read, “geo-epidemiological studies….”**

Thank you, this word was corrected.

**11. Lines 93-94: It should read, “infectious diseases, and other conditions,……….health policies and knowledge of disease spread.”**

Thank you, this line was corrected.

**12. Line 95: Replace “derive” with “measure”.**

Thank you, this word was corrected.

**13. Line 99: Replace “for such a task” with “to accomplish these objectives”.**

Thank you, this line was corrected.

**14. Line 101: Replace “risks” with “risk”.**

Thank you, this word was corrected.

**15. Line 104: Replace “considering” with “concerning” and “in Mexico” with “of Mexico”.**

Thank you, these words were corrected.

**16. Line 116: It should read, “used three age groups for subsequent analyses:”**

Thank you, this line was corrected.

**17. Line 130: Replace “are” with “were”.**

Thank you, this word was replaced.

**18. Line 131: Replace “by” with “for”.**

Thank you, this preposition was corrected.

**19. Line 147: Replace “dying” with “mortality” when you first define “MRt”.**

Thank you, this word was corrected.

**20. Lines 152: Replace “are” with “were”.**

Thank you, this verb was corrected.

**21. Table 1: Please make certain the number of decimal places presented is consistent with the precision the variable was measured. Technically, the natural log of the number of tested individuals will be your offset in subsequent Poisson regression models; it may be better to comment on this being used for the offset in a footnote rather than the column heading.**

We checked how each variable should be reported in terms of number of decimal places. Only two columns (death and number of tested individuals) are non-negative integers, the remaining are percentages or variables in which decimal places should be used. However, after checking the table, we realized that Table 1 (Continued) contained only percentages, but that three, instead of two decimal places were used there. We corrected this error.

We also eliminated the word offset in the column heading corresponding to number of tested individuals and added a footnote indicating that this column corresponds to an offset.

**22. Line 168: Replace “along the territory” with “throughout the country” or “among the states of Mexico”.**

Thank you, this line was corrected.

**23. Lines 184-185: Replace “a one” with “one”.**

Thank you, this word was modified.

**24. Line 204: Replace “states” with “the centroids of states”.**

Thank you, this line was corrected.

**25. Lines 215-240: Replace each “,” between numbered phrases with a “;”. This type of punctuation will make it easier for readers to follow the long number of complex phrases.**

Thank you, this punctuation was modified in all the paragraph.

**26. Line 232: Do the authors mean “after controlling for the confounders”? Please edit accordingly if this is correct.**

It is correct the Reviewer’s assumption; thus, we modified this phrase.

**27. Line 233: It should read, “the other three models…”**

We think that if we directly apply this change the meaning of this line is not correct. Here, we mean that three Poisson models are fitted, one for each variable (ICU, intubated, and hospitalization) used as outputs. We think it is important to explain that these are Poisson linear models since again the responses are percentages. Hence, we did not eliminate the word “Poisson”, but eliminated the word “other”. We added that they are used as outputs in these regressions to better clarify this part.

**28. Line 261: Replace “superior one” with “higher quartile”.**

Thank you, this line was corrected. Concerning Figure 1, we observed that in parentheses we had included “risks above 3.5%” without specifying that they corresponded to the raw values (adjusted for sex and age) and not to the smoothed values. Thus, we modified what was included in parentheses (Line 264).

**29. Line 265: There is an inconsistency between your text where Moran’s I = -0.059 and your figure where Moran’s I = -0.054.**

Thank you. The text was incorrect, the value corresponded to that before adjusting for sex and age. We corrected this error.

**30. Line 295: Why include the outcome in the matrix of correlations? Multicollinearity is only an issue for the independent variables. I would remove the dependent variable from the matrix unless the authors can provide an explanation for this decision to avoid confusing readers.**

We included the response to identify how each explanatory variable and confounder were separately associated with the response. However, as this paragraph was written, it only corresponded to multicollinearity and nothing was discussed in Results or Discussion about these univariable associations with the response. Hence, to avoid adding more sentences explaining these associations, we decided that, as suggested by the Reviewer, it was better just to eliminate the risk (response) from the correlation plot. The paragraph in which Figure 3A is referred was modified accordingly.

Additionally, we deeply apologize, but we have realized that the version of Figure 3 we uploaded by mistake in the journal system for the last correction, which corresponds to the third version of the manuscript, was the one in the original (first) version of the manuscript, instead of the one corresponding to the second version (the correlation plot in the first version did not include the age groups we finally used). For the second version no additional changes were suggested by the Reviewers affecting this Figure; thus, this error does not affect anything. This is also relevant because there is a comment below about the map for the residuals corresponding to the geographically weighted regression, which is contained in Figure 3B, and this map was also incorrect. For this last corrected version, we will be certain that all uploaded figures are the correct ones.

**31. Lines 307-312: I would suggest the authors review Methods in Epidemiologic Research by Dohoo et al. concerning assessing the fit of Poisson regression models (**[**https://projects.upei.ca/mer/**](https://projects.upei.ca/mer/)**). They may want to fit a negative binomial regression model for their global model to assess if there is really an over-dispersion issue (i.e., assess if alpha is significantly different from zero).**

Thank you. We are aware that a negative binomial model is an alternative to a quasi-Poisson model; and that, to consider possible overdispersion the response is assumed to have a Poisson distribution conditional to a parameter that is gamma distributed, resulting this in a negative binomial distribution. Under this negative binomial distribution, the dispersion depends on the mean of the response plus a term corresponding to the product of the squared mean of the response and a non-negative parameter named gamma (or alpha according to the Reviewer). Thus, the second part in this sum corresponds to how much the dispersion of the response is increased when compared with the mean. We fitted this negative binomial model, and the estimated parameters were similar as those obtained fitting the Poisson regression model as shown below. The dispersion parameter gamma (or alpha) corresponds to 1/Theta, where Theta is a value provided in the R output. This estimated value was of almost zero (0.0000065) and not significantly different from zero (p-value=0.5). For this test, we used a likelihood ratio test (LRT) comparing a nested model, corresponding to the Poisson regression model, with the negative binomial regression model; thus, the null hypothesis corresponds to H0: gamma = 0. Since the null hypothesis involves the lower bound associated with the gamma parameter (zero), a Chi-squared distribution with one degree of freedom is used and the corresponding p-value is divided by two to obtain the correct p-value for the test, e.g. (Agresti, 2015, Foundations of Linear and Generalized Linear models, p. 249-250). According to this analysis there was no significant overdispersion when considering the negative binomial distribution.

For completeness, we decided to include both forms to analyze overdispersion, a quasi-Poisson model, with the corresponding over dispersion parameter, and the analysis considering a negative binomial distribution.

```
Negative binomial model

Coefficients:
                    Estimate Std. Error z value Pr(>|z|)
(Intercept)         -5.37578    1.27311  -4.223 2.42e-05 ***
Diabetes             0.14137    0.03336   4.238 2.25e-05 ***
Obesity              0.11534    0.01930   5.975 2.30e-09 ***
ICU (residuals)      0.20086    0.02536   7.920 2.37e-15 ***
GDP                  0.26522    0.04308   6.156 7.44e-10 ***
Internal migration   0.23679    0.03516   6.735 1.63e-11 ***
External migration   0.29915    0.12647   2.365  0.01801 *
Age group 10-39     -0.09675    0.01983  -4.878 1.07e-06 ***
Intubated (residual) 0.19779    0.03306   5.983 2.19e-09 ***
Physicians’ ratio    0.62382    0.07053   8.845  < 2e-16 ***
Cardio vascular     -0.05014    0.07065  -0.710  0.47792
Hospitalization(res.)-0.04262   0.01641  -2.598  0.00937 **
---
Signif. codes:  0 ‘***’ 0.001 ‘**’ 0.01 ‘*’ 0.05 ‘.’ 0.1 ‘ ’ 1


Poisson model

Coefficients:
                    Estimate Std. Error z value Pr(>|z|)
(Intercept)         -5.37567    1.27300  -4.223 2.41e-05 ***
Diabetes             0.14137    0.03335   4.239 2.25e-05 ***
Obesity              0.11534    0.01930   5.975 2.29e-09 ***
ICU (residuals)      0.20086    0.02536   7.921 2.36e-15 ***
GDP                  0.26523    0.04308   6.157 7.41e-10 ***
Internal migration   0.23680    0.03515   6.736 1.62e-11 ***
External migration   0.29916    0.12645   2.366  0.01799 *
Age group 10-39     -0.09675    0.01983  -4.879 1.07e-06 ***
Intubated (residual) 0.19779    0.03305   5.984 2.18e-09 ***
Physicians’ ratio    0.62382    0.07051   8.847  < 2e-16 ***
Cardio vascular     -0.05013    0.07065  -0.710  0.47797
Hospitalization(res.)-0.04263   0.01640  -2.599  0.00936 **
```

**32. Lines 313-314: It should be clear in the methods which interactions were tested.**

Thank you. We have added exactly which interaction effects were tested in the Methods section (Lines 316-321).

**33. Lines 321-322: Based on the legend, the largest residuals should be in yellow and black, but these do not appear to be the colours of Veracruz and Yucatan in Figure 3b. Please re-check that these regions have the largest residuals.**

As mentioned in 30, we are sorry, but by mistake we uploaded the last time Figure 3 corresponding to the first (original) version of the manuscript. In that version, the spatial model was different, and consequently the residuals, when compared with the model in the second version, the one the Reviewers already revised in which the correct Figure 3 was included. In the correct model, Veracruz and Yucatan were the states with the lowest residuals (in blue) with values less than -2 (-2.42 for Veracruz and -2.14 for Yucatan), whereas the largest positive value is less than 1.5 (value of 1.46). We have now carefully checked that each of the uploaded figures is correct.

**34. Table 2: Please clarify if these are 95% confidence intervals in the column heading. Usually the terms “upper” and “lower” confidence intervals are used rather than “inferior” and “superior”.**

Thank you. We have clarified that we calculated 95% confidence intervals and changed the terms used for the interval limits.

**35. Line 373: Replace “overall” with “throughout”.**

Thank you, this word was corrected.

**36. Line 376: Replace “in” with “on” and be consistent with capitalizing the word “north” (see line 373).**

Thank you very much. We replaced the word and capitalized this word. The same capitalization was applied for all those cardinal points referring to regions in Mexico in the manuscript.

**37. Line 383: Replace “along” with “throughout”.**

Thank you, this word was corrected.

**38. Lines 384-385: It might be clearer and more succinct to write, “and how the associations with some independent variables varied among states.”**

In this sentence we wanted to include two things. The first one is the type of association (positive or negative) between independent variables and the response. The second one corresponds to the variation of these associations among states. We modified this sentence to include both parts according to the Reviewer’s suggestion.

**39. Line 388. It might be better to write, “in the early stages of the outbreak.”**

Thank you, the word “moments” was replaced by “stages”.

**40. Lines 389-390: It should read, “an increase in…….include variables…..”**

Thank you, this line was corrected as suggested.

**41. Line 392: Replace “greatest” with “largest”.**

Thank you, this word was corrected.

**42. Lines 407-408: It might be better to write, “related to the economic activities of these states that prevent adequate self-isolation measures and, unlike the states with the largest cities, do not have the health infrastructure to prevent deaths among clinical cases.”**

Thank you very much, we added this part. The paragraph has greatly improved with this suggestion.

**43. Line 389: Replace “on” with “in”.**

Thank you, this line word corrected.

**44. Line 390: Replace “percentages” with “variables”.**

Thank you, this word was corrected.

**45. Line 392: Replace “greatest” with “largest”.**

Thank you, this word was corrected.

**46. Lines 415-416: It should read, “at least until the period of our study, there was no significant clustering of states…….”**

Thank you, this line was corrected.

**47. Line 418: Replace “suggests” with “might explain the higher…..”**

Thank you, this word was replaced.

**48. Line 419: Replace “on such” with “in this”.**

Thank you, these words were replaced.

**49. Line 420: It should read, “most strongly associated with the levels of diabetes, internal migration, and the physician-to-population ratio.”**

Thank you, this line was corrected.

**50. Line 422: Do the authors mean, “a particularly strong positive association…”?**

Yes, we meant what the Reviewer has asked; thus, we modified this sentence accordingly.

**51. Line 424: Replace “correspond” with “corresponded”.**

Thank you, this word was corrected.

**52. Line 425: Replace “urbanity” with “urbanicity”.**

Thank you, this word was corrected.

**53. Line 426: Drop “which”.**

Thank you, this word was eliminated and the sentence adjusted accordingly.

**54. Line 427: It should read, “but it has a relatively similar strength of association across all of Mexico.”**

Thank you, this line was corrected.

**55. Line 433: Replace “little” with “slightly”.**

Thank you, this word was replaced.

**56. Line 434: It should read, “The associations have also….”**

Thank you, these words were corrected.

**57. Line 437: Replace “on” with “throughout”.**

Thank you, this word was corrected.

**58. Line 439: Replace “an” with “the”.**

Thank you, this word was corrected.

**59. Line 441: Replace “, being” with “since the state is”.**

Thank you, this line was corrected.

**60. Line 445: Replace “being” with “making”.**

Thank you, this word was corrected.

**61. Lines 447-455: It should read, “in the early stages of the pandemic, which is the focus of our analysis, and this would have resulted in many issues concerning model fit and convergence. To obtain similar results, we would have required different tools, such as zero-inflated geographical models, that are not currently available. Future studies could focus on the characteristics of municipalities with zero cases and mortalities, and focus on the spatio-temporal analysis of these COVID-19 data.”**

Thank you. We have modified this paragraph according to the suggested lines.

**62. Line 456: It should read, “models fitted since most of the statistical…..”**

Thank you, this line was corrected.

**63. Line 457: Replace “is” with “was”.**

This line was modified according to the results obtained from both the quasi-Poisson and negative binomial models, the latter was fitted for this revised version.

**64. Line 464: It might sound better to state, “the same issue was present for age distribution.”**

Thank you, this line was corrected.

**65. Line 470-471: It should read, “or individuals due to the potential for ecological bias.”**

Thank you, this line was corrected.

**66. Line 477: It should read, “variables, such as……….diseases, cannot……”**

Thank you, this line was corrected.

**67. Lines 479-482: It should read, “could help in the identification of public policies that could limit the impact of future epidemics. Even though our study focused on the early stages of SARS-CoV-2 spread, its results allow us to………………………………should be taken to control additional waves of COVID-19 or………….”**

Thank you, this line was corrected.

**References:**

**68. Make certain to not use capital letters for the first letter of each word in manuscript titles for the following references: 4, 5, 22, 33, 34.**

Thank you, we have modified these references.

**69. Line 533: It should read, “SARS-CoV-2”.**

Thank you, we have corrected this word.

We also updated reference [30] that has been published. We corrected reference [19], since it corresponds to a technical report, and we realized that the web page we mentioned before did not include the corresponding pdf file. Similarly, for reference [35].

7. PLOS authors have the option to publish the peer review history of their article ([what does this mean?](https://journals.plos.org/plosone/s/editorial-and-peer-review-process#loc-peer-review-history)). If published, this will include your full peer review and any attached files.


If you choose “no”, your identity will remain anonymous but your review may still be made public.


**Do you want your identity to be public for this peer review?** For information about this choice, including consent withdrawal, please see our [Privacy Policy](https://www.plos.org/privacy-policy).

Reviewer #2: No
